# Blocking interleukin-23 ameliorates neuromuscular and thymic defects in myasthenia gravis

**DOI:** 10.1186/s12974-023-02691-3

**Published:** 2023-01-13

**Authors:** José A. Villegas, Jérôme Van Wassenhove, Judith Merrheim, Karen Matta, Samy Hamadache, Clémence Flaugère, Pauline Pothin, Frédérique Truffault, Sébastien Hascoët, Nicola Santelmo, Marco Alifano, Sonia Berrih-Aknin, Rozen le Panse, Nadine Dragin

**Affiliations:** 1 Inserm, Institut de Myologie, Centre de Recherche en Myologie, Sorbonne Université, 105 Bd de l’hôpital, 75013 Paris, France; 2 Hôpital Marie Lannelongue, Le Plessis-Robinson, France; 3 Chirurgie Thoracique des deux Rives, Rhéna - Clinique de Strasbourg, Strasbourg, France; 4 Department of Pathology, Cochin University Hospital Group, AP-HP, Paris-Descartes University, Paris, France

**Keywords:** Autoimmunity, Inflammation, Neuromuscular junction, Germinal centers, Th17, Muscle regeneration

## Abstract

Acetylcholine receptor (AChR) myasthenia gravis (MG) is a chronic autoimmune disease characterized by muscle weakness. The AChR^+^ autoantibodies are produced by B-cells located in thymic ectopic germinal centers (eGC). No therapeutic approach is curative. The inflammatory IL-23/Th17 pathway is activated in the thymus as well as in the blood and the muscle, contributing to the MG pathogenic events. We aimed to study a potential new therapeutic approach that targets IL-23p19 (IL-23) in the two complementary preclinical MG models: the classical experimental MG mouse model (EAMG) based on active immunization and the humanized mouse model featuring human MG thymuses engrafted in NSG mice (NSG-MG). In both preclinical models, the anti-IL-23 treatment ameliorated MG clinical symptoms. In the EAMG, the treatment reduced IL-17 related inflammation, anti-AChR IgG2b antibody production, activated transduction pathway involved in muscle regeneration and ameliorated the signal transduction at the neuromuscular junction. In the NSG-MG model, the treatment reduced pathogenic Th17 cell population and expression of genes involved in eGC stabilization and B-cell development in human MG thymus biopsies. Altogether, these data suggest that a therapy targeting IL-23p19 may promote significant clinical ameliorations in AChR^+^ MG disease due to concomitant beneficial effects on the thymus and skeletal muscle defects.

**Supplementary Information:**

The online version contains supplementary material available at 10.1186/s12974-023-02691-3.

## Background

Myasthenia gravis (MG) is a rare autoimmune pathology mainly due to antibodies directed against the acetylcholine receptor (AChR) at the neuromuscular junction (AChR^+^ MG). MG is often associated with thymic alterations such as follicular hyperplasia or thymoma. Hyperplastic AChR^+^ MG thymuses harbor a chronic loop of inflammation. To date, there is no cure for AChR^+^ autoimmune MG and symptomatic treatments rely on different types of nonexclusive therapies. The first-line treatment includes symptomatic therapies with anticholinesterase drugs that enable rapid relief of the clinical symptoms but have no effect on the immunopathology [1, 2]. The second line of therapy is long-term treatment with corticosteroids and alternative immunosuppressors [3]. Although these therapies are effective in a considerable number of patients, they are clinically challenging due to their multiple side effects [3, 4]. In addition, patients with MG crisis may undergo plasma exchange or receive intravenous immunoglobulins. Another therapeutic option is thymectomy combined with a progressive decreased level of corticosteroids, an effective therapy to ameliorate MG symptoms [5] but that does not provide a cure and several patients are reluctant to surgery. Hence, the need for novel therapies that can alleviate the symptoms remains a priority.

As of October 2022, there are 48 registered clinical trials in phase 2 or 3 linked to myasthenia gravis, listed by the clinicaltrials.gov website. More recently, the American Food and Drug Administration has approved an Fc receptor blocker (efgartigimod alfa-facab) and a C5 complement inhibitor (ravulizumab) for the treatment of generalized AChR^+^ MG patients [6, 7].

Thymuses from early-onset AChR^+^ MG patients are characterized by B cell infiltration often associated with ectopic germinal centers (eGCs) [8], which are source of autoreactive B cells producing anti-AChR antibodies [9]. AChR^+^ MG thymuses displaying follicular hyperplasia harbor upregulated expression of B-cell chemoattractant CXCL13 and CCL21 [10, 11] and cytokines such as IFN-β, IL-1β, IL-6, and TGF-β1 [12, 13]. This inflammatory environment is associated with a clear imbalance between regulatory and Th17 cells, in favor of the latter [14, 15]. Th17 cells differentiate and maturate in a two-step process induced by the cytokine environmental context. The differentiation step involves IL-6 and TGF-β1, and the activation step is induced by IL-23 and TGF-β3 [16]. We have shown previously that the IL-23/T helper 17 (Th17) pathway is activated and participates in the perpetuation of thymic inflammatory status in AChR^+^ MG [17]. Indeed, medullary thymic epithelial cells (mTECs) overexpress IL-23, in addition to IL-1β, IL-6 and TGF-β1, promoting the differentiation of pathogenic Th17 cells. Moreover, a loop exists in which overactivated Th17 cells sustain IL-23 overexpression by MG mTECs [17]. Finally, IL-23 level is also significantly increased in the serum of AChR^+^ MG patients compared to healthy controls [17].

The IL-23/Th17 cell pathway is critical in the development of several autoimmune diseases such as multiple sclerosis, psoriasis and rheumatoid arthritis. In experimental autoimmune encephalomyelitis (EAE), a mouse model of multiple sclerosis, IL-23-differentiated Th17 cells overexpress IL-17 and podoplanin and promote the formation of eGCs [18]. Moreover, IL-23-differentiated Th17 cells may overexpress IL-21, an inflammatory cytokine that regulates the expression of β-galactoside α 2,6-sialyltransferase 1 (ST6gal1) [19]. Of note, ST6gal1 is an enzyme that transfers sialic acid to glycoproteins such as immunoglobulins (IgGs) and cell surface glycoproteins. Consequently, ST6gal1 modulates antibody production and pathogenicity [20], and cell differentiation and proliferation [21]. In addition to their roles in IgG production, Th17 cells sustain the activation of inflamed stromal cells in lymph nodes. Hence, pathogenic Th17 cells and their related cytokines are considered new potential therapeutic targets for alleviating and treating various autoimmune diseases [22]. For instance, ongoing clinical trials show that blocking different factors of the IL-23/Th17 pathway can ameliorate inflammatory pathologies such as psoriasis or rheumatoid arthritis [23]. Hence anti-IL-23 treatment (guselkumab) has proven efficacious in ameliorating psoriasis-related symptoms in affected patients [24].

IL-23 is a dimeric cytokine composed of two subunits, IL-23p19 and IL-12p40, a subunit shared with IL-12. Here, we investigated the therapeutic potential of blocking IL-23p19 in autoimmune MG using preclinical mouse models. The classical EAMG mouse model has been largely used to determine the mechanisms behind MG development by using genetically modified mice. For instance, Wei Wang et al. showed that IL-12/IL-23p40 and IFN-γ double-KO mice are susceptible to EAMG development [25]. Moreover, IL-17 deficient mice are protected from EAMG development and display a reduced production of anti-T-AChR antibodies [26]. These reports suggest that Th17 cells may contribute to the development of MG. The classical EAMG model is pertinent to investigate the impact of the anti-AChR attack at the neuromuscular junction and associated muscle weaknesses. However, this model does not show any of the thymic abnormalities observed in the human disease. In contrast, the NSG-MG model allows to analyze and decipher the pathological mechanisms occurring in the human thymus. NSG mice are engrafted with human AChR^+^ MG thymus fragments. This humanized model presents different human MG features, including increased inflammatory markers (T cells and cytokines) in the engrafted thymus, in the blood and in the spleen and anti-human AChR autoantibodies leading to myasthenic muscle manifestations [27].

To improve the MG outcome, we investigated the effect of an anti-IL23 therapy in these two relevant experimental mouse models of MG. We evaluated the signs of ameliorations at different levels such as thymic inflammation, autoantibody production and associated muscle weaknesses.

## Methods

### Study approval for human samples

Control and MG human thymuses were obtained from patients undergoing cardiac surgery or thymectomy at the hospital Marie Lannelongue Chirurgical Center (Le Plessis-Robinson, France), the Strasbourg Civil Hospital (Strasbourg, France), and Cochin University Hospital group (Paris, France). Five MG patients aged 22 to 26 years old with anti-AChR antibodies but without thymoma were included. The patients were stabilized before the thymectomy. They have different severity scores and were on ocular stage, however they have gone through generalized disease stage.

### Study approval for mouse models

Mouse models, experiments and the group size were approved by the French Ministry of Agriculture Committee for Animal Use (authorization numbers #24755, #3692-2016012111336184 and #02622-22). All animals were handled according to the Animal Care and Use of Laboratory Animal guidelines and in a facility approved by the French Ministry of Research (authorization number B-75-13-20).

### EAMG mouse model

Five-week-old female C57BL6 mice were obtained from Janvier Laboratories (Le Genest Saint-Isle, France) and stabulated in our animal facility for 1 week. Briefly, mice were injected subcutaneously in hind footpads and in the back with an emulsion containing purified torpedo acetylcholine receptor (T-AChR) (30 μg), complete Freund’s adjuvant (CFA) (Sigma, Saint Quentin Fallavier, France) and nonviable Mycobacterium tuberculosis (1 mg/mouse) (BD Difco, Villepinte, France). Control mice were injected with an emulsion containing only CFA and nonviable Mycobacterium tuberculosis. Three weeks after the first immunization, mice received a second immunization with a solution containing CFA and T-AChR, or CFA only [28]. EAMG mice were treated by intraperitoneal injection with 100 μg of monoclonal anti-mouse IL-23p19 antibody (clone G23-8, Thermo-Fisher, Courtaboeuf, France). Treatment or placebo (NaCl) were started 2 weeks after the second immunization with a weekly dose and continued for either 2 or 4 weeks. One week after the last treatment, the mice were euthanized. Blood, spleen and muscle (tibialis anterior) were recovered and used freshly or stored at − 80 °C for later analysis.

### NSG-MG mouse model

NOD-SCID IL-2Rγ-null (NSG) mice were obtained from Charles Rivers laboratories and kept in our facility under specific pathogen-free conditions. Mice aged 8–12 weeks were subcutaneously engrafted with freshly collected human thymic biopsies through a minimal incision. We followed the protocol described and established by Sudres et al. [27]. Since no difference in disease development was observed in the NSG-MG model when biopsies from prednisolone-treated MG patients were used, we engrafted thymic biopsies regardless of patient history of prednisolone [27]. NSG-MG mice were treated by intraperitoneal injection with 100 μg/mouse/week monoclonal anti-human IL-23p19 antibody (clone HNU2319, Thermo Fisher, Courtaboeuf, France). Treatment was given for 4 weeks, starting at day 15 after engraftment which is also the day on which clinical MG manifestations arise [27]. Control mice were treated with an equivalent volume of physiological serum (Ab diluent). On day 42 after grafting, mice were euthanized, and the remnants of xenogeneic human thymuses, the spleen, blood, and tibialis anterior muscle were recovered and used freshly or stored at − 80 °C for later analyses.

### Clinical tests

A clinical analysis of each mouse was performed weekly as previously described [28]. Animals were weighed and checked for signs of fatigue or unusual behavior (ears and/or tail down, abnormal movements or reduced mobility). To determine the clinical disease score, we performed the grip test (after treadmill exercise) and the hire test. The different tests combined together provided a disease score between 0 (not sick) and 9 (dead or euthanized before the end of the experiment), as fully detailed in Weiss et al. [28].

### Electromyography

Groups have used electromyography analysis to assess impaired neuromuscular transmission in EAMG model. Meinen et al. used low-frequency repetitive nerve stimulation of the sciatic nerve [29]. Therefore, electromyography was performed as previously described [30] by the Sorbonne University platform AniFM—Evaluation de la fonction musculaire chez le petit animal. The compound muscle action potential (CMAP) in response to nerve stimulation, maximal muscle force, and muscle relaxation time were evaluated at the time of sacrifice.

### RNA extraction and reverse transcription

Frozen human thymus, mouse spleen and tibialis anterior muscle were homogenized with the FastPrep FP120 instrument (Qbiogen, Illkirch, France). Total RNA was extracted from the thymus, spleen and muscle samples using a TRIzol RNA isolation kit (Invitrogen, Cergy-Pontoise, France). Total mRNA (1 μg) was reverse transcribed using the AMV first-strand cDNA Synthesis Kit (Roche Diagnostics, Meylan, France) according to the manufacturer’s instructions.

### Quantitative real-time PCR

Gene expression was evaluated by quantitative real-time PCR performed using a Light-Cycler apparatus (Roche Diagnostics; Meylan, France) as previously described [31]. Each PCR was performed using the Fast-start DNA Master SYBR Green I Kit (Roche Diagnostics; Meylan, France) according to the manufacturer’s instructions. Each cDNA sample was run in duplicate. Samples were normalized as specified in the figure legends. The list of primers is summarized in Table 1.

**Table 1:** List of the primers used in the study

Gene		Forward	Reverse
Human	*AID*	AAGGGCTGCATGAAAATTCAGT	CGTCTCGTAAGTCATCAACCTC
	*Blimp1*	AAGCAACTGGATGCGCTATGT	GGGATGGGCTTAATGGTGTAGAA
	*CXCL13*	CTCTGCTTCTCATGCTGCTG	TGAGGGTCCACACACACAAT
	*GAPDH*	CGACCACTTTGTCAAGCTCA	AGGGGTCTACATGGCAACTG
	*GM-CSF*	TCCTGAACCTGAGTAGAGACAC	TGCTGCTTGTAGTGGCTGG
	*IFN-γ*	TCCCATGGGTTGTGTGTTTA	AAGCACCAGGCATGAAATCT
	*IL-17A*	CCCCTAGACTCAGGCTTCCT	AGTTCATTCTGCCCCATCAG
	*IL-6*	TGAGGTGCCCATGCTACATTT	TCTGCGCAGCTTTAAGGAGTT
	*Keratin 14*	TTCTGAACGAGATGCGTGAC	GCAGCTCAATCTCCAGGTTC
	*Ki67*	AAGCCCTCCAGCTCCTAGTC	TCCGAAGCACCACTTCTTCT
	*Podoplanin*	TGTGGCGCTTGGACTTTGT	GTGTAACAGGCATTCGCATCG
	*ST6gal1*	TGCAGCCTCACGACAGATAC	ACCCTGAGAGACCTTCAGCA
Mouse	*Aid*	CCAGACTTTGGGTCGTGAAT	TGGCTTGTGATTGCTCAGAC
	*Cypa*	CACCGTGTTCTTCGACATCAC	CCAGTGCTCAGAGCTCGAAAG
	*Gapdh*	AACTTTGGCATTGTGGAAGG	ACACATTGGGGGTAGGAACA
	*Il-17a*	TCTCTGATGTTGCTGCT	CGTGGAACGGTTGAGGTAGT
	*Il-6*	AGTTGCCTTCTTGGGACTGA	TCCACGATTTCCCAGAGAAC
	*Il-6R*	GACTATTTATGCTCCCTGAATGATCA	ACTCACAGATGGCGTTGACAAG
	*Il-21*	GGACCCTTGTCTGTCTGGTAG	TGTGGACGTGATAGAAGTTCAGG
	*MyoD*	AAGACGACTCTGACGGCTTG	TCTGGTGAGTCGAAACACGG
	*MyoG*	GGGCAAACTCAGGAGCTTCT	CAGAGGCTTTGGAACCGGAT
	*Pax7*	GGGCTCTTCAAGGTCTGGAC	CAGGGAGCAAGGAATGTGGA
	*Podoplanin*	GCCAGTGTTGTTCTGGGTTT	AGAGGTGCCTTGCCAGTAGA
	*Sdf1*	GCTCTGCATCAGTGACGGTA	ATTTCGGGTCAATGCACACT
	*St6gal1*	CCTTATGCGGGCAATAGAAA	ACTTCCTATGCACCGTGGAC
	*Tgf-β1*	CAAGGGCTACCATGCCAACT	CCGGGTTGTGTTGGTTGTAGA
	*Tgf-β3*	GAGACCGGATAGCGAGTGGA	TTGGCCGCCTCTAACGATAT

### Human cytokine ELISA

The levels of the human cytokines IL-2, IL-6, IL-17, IL-21, TGF-β1 and TGF-β3 were evaluated in extracted protein samples. Total tissue proteins were extracted with a solution containing 5% Tris HCl 20 mM, 0.1% Triton X100, and one tablet of protease inhibitor cocktail (Roche-Diagnostics, Meylan, France) using a fast prep apparatus. All ELISA kits were from R&D Systems (Lille, France). Each ELISA was performed in duplicate and according to the manufacturer's instructions. ELISA reactions were read with a SPARK ELISA microplate reader (Tecan, Männedorf, Switzerland).

### Detection of human IgG and anti-AChR antibodies

Quantification of total circulating human IgG in NSG mice was performed by ELISA. Polyclonal rabbit anti-human IgG was incubated overnight in 96-well plates at 4 °C. Next, blocking buffer (1% PBS–BSA) was added and incubated for 1 h at room temperature, followed by two PBS wash steps. Then, 100 μl of serum and standards were added and incubated for 1 h. Next, HRP-coupled polyclonal rabbit anti-human IgG was added and incubated for 2 h at 37 °C. The results were visualized with the addition of 3,3′,5,5′ tetramethylbenzidine (Sigma-Aldrich, Lyon, France). The reaction was stopped with H_3_PO_4_. Absorbance was measured at 450 nm. The antibodies used to detect human total IgG were purchased from DAKO (Courtaboeuf, France).

Detection of human anti-AChR antibodies in engrafted NSG mouse serum was performed using the AChR Autoantibody ELISA Kit from RSR (RSR Limited, Cardiff, United Kingdom) according to the manufacturer’s instructions.

### Detection of anti-T-AChR antibodies

Ninety-six-well ELISA plates were coated overnight at 4 °C with 1 μg/ml T-AChR diluted in 10 mM NaHCO3 buffer, pH 9.6. Then, the ELISA plates were blocked with 10% FCS in PBS at 37 °C for 2 h. Then, 100 µl of mouse serum (dilution 1/100,000) were added and incubated for 2 h at 37 °C. After washing with PBS–Tween buffer, we completed the sandwich ELISA by adding 100 µl of biotinylated anti-mouse IgGs (total IgG, IgG1 or IgG2b) (dilution 1/10,000) (Dako, Courtaboeuf, France). The plates were incubated for 2 h at 37 °C and then washed and incubated with 100 µl of streptavidin-HRP (Dilution 1/20,000) (Life Technologies, Courtaboeuf, France) for 30 min, and the results were visualized with the addition of tetramethylbenzidine. Optical density was determined at 450 nm in a SPARK ELISA microplate reader (Tecan, Männedorf, Switzerland).

### Flow cytometry analysis

To analyze the circulating human cells in NSG mice, fresh blood was taken once a week. Lymphocytes were obtained after lysis of red blood cells with BD lysis buffer (BD Biosciences, Le Pont de Claix, France) for 10 min and stained with anti-human antibodies (BD Biosciences, Le Pont de Claix, France). The antibodies used for this analysis are listed in Table 2. Cells were acquired using a FACS Canto II Analyzer (BD Biosciences, Le Pont de Claix, France) and analyzed using FlowJo software (Tree Star, Olten, Switzerland).

**Table 2:** List of the antibodies used for flow cytometry (FC) and immunohistochemistry (IHC) analyses

Antibody target	Conjugate	Host	Reactivity	Clone	Supplier	Used in
CCR4	PE	Mouse	Human	205410	R&D	FC
CCR6	APC	Mouse	Human	11A9	BD Bioscience	FC
CD127	FITC	Mouse	Human	ebioRDR5	eBioscience	FC
CD19	FITC	Mouse	Human	HIB19	eBioscience	FC
CD25	PE	Mouse	Human	BC96	eBioscience	FC
CD4	V450	Mouse	Human	RPA-T4	BD Bioscience	FC
CD45	V500	Mouse	Human	H130	BD Bioscience	FC
CD8	ACP-H7	Mouse	Human	SK1	BD Bioscience	FC
IL-23R	PerCP	Mouse	Human	218213	R&D Systems	FC
Keratin 5	Purified	Rabbit	Human	Poly19055	BioLegend	IHC
Keratin 14	Purified	Rabbit	Human	Poly19053	BioLegend	IHC
CCR6	APC	Rat	Mouse	140706	BD Pharmingen	FC
CD127	V450	Rat	Mouse	SB199	BD Bioscience	FC
CD25	FITC	Rat	Mouse	16184	BD Bioscience	FC
CD31	Purified	Rat	Mouse	MEC 13.3	BD Pharmingen	IHC
CD4	APC-eF780	Rat	Mouse	GK 1.5	eBioscience	FC
CD62L	PerCP Cy5.5	Rat	Mouse	MEL14	eBioscience	FC
CD8	PE Cy7	Rat	Mouse	53–6.7	BD Bioscience	FC
Anti-rabbit IgG	Alexa Fluor 594	Chicken	Rabbit	N/A	Invitrogen	IHC
Anti-rat IgG	Alexa Fluor 488	Chicken	Rat	N/A	Invitrogen	IHC

At sacrifice, remnants of xenogeneic human thymuses and mouse spleens were mechanically dissociated in PBS to obtain a cell suspension labeled as described above for the blood cells.

### Immunohistochemistry analysis

Cryostat sections (7 μm thick) of xenogeneic human thymuses were fixed with acetone and dried for 1 h. Slides were pre-incubated with blocking buffer (PBS, 0.1% BSA, 10% FBS, 0.3 M glycine, and 1% Tween) for 1 h at room temperature. Then, they were incubated overnight at 4 °C with antibodies raised against human or mouse antigens. Secondary labeling was done using Alexa 488- and Alexa 594-coupled IgG antibodies raised in chicken or donkey. Images were acquired with a Zeiss Axio observer Z1 inverted microscope using 20× magnification (Carl Zeiss, Le Pecq, France). The antibodies used for this analysis are listed in Table 2.

### Statistical analysis

Nonparametric tests (Wilcoxon test for paired data, Mann–Whitney t-test or ANOVA for unpaired values) were used to compare groups as specified in each figure legend. Values were reported as the mean ± SEM. GraphPad Prism software was used to generate the graphs and to perform the statistical analysis. Statistical significance was recognized at *P* < 0.05.

## Results

### In the EAMG model, anti-IL-23p19 treatment ameliorates MG clinical manifestations

**Fig. 1:**
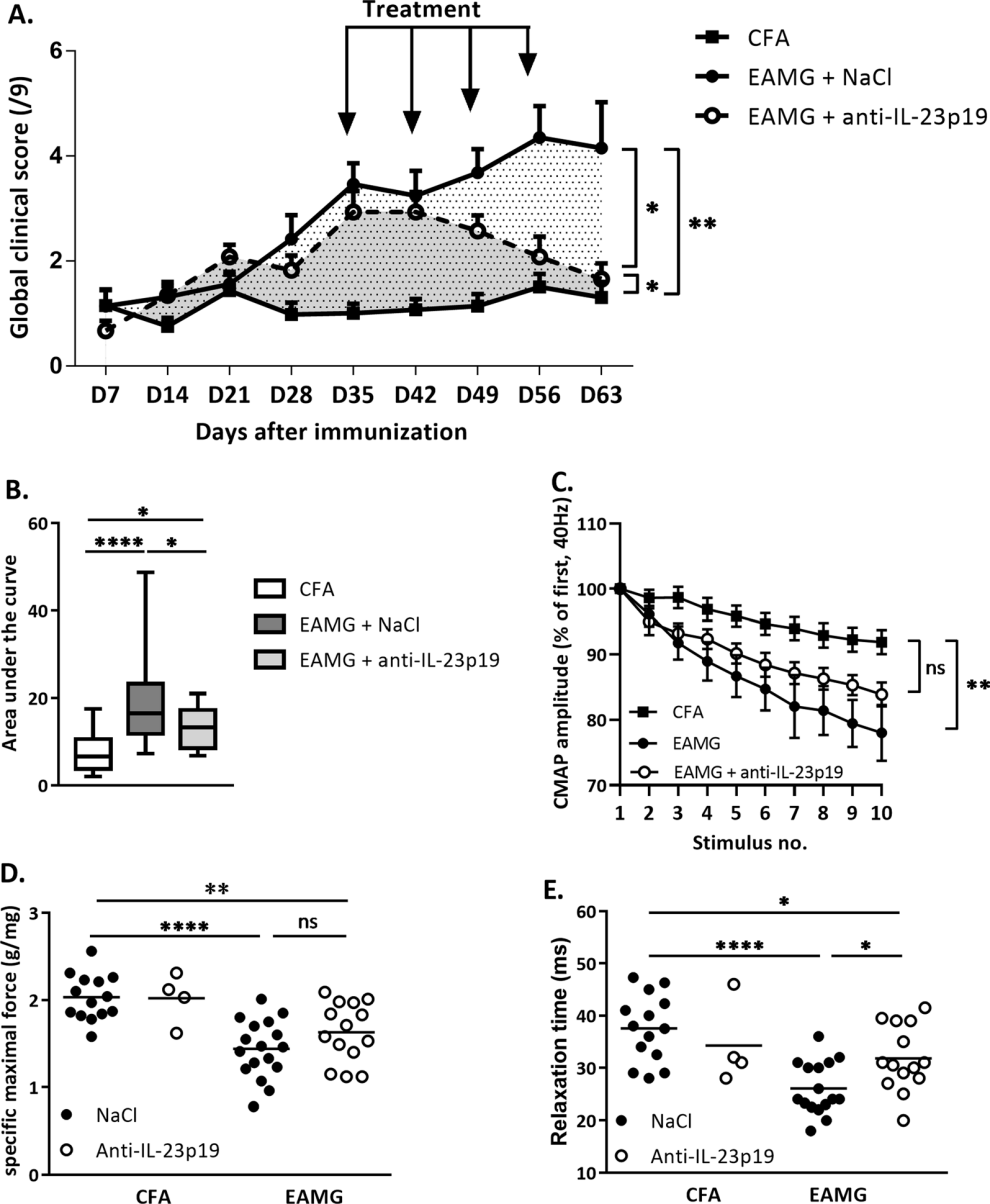
Effects of anti-IL-23p19 treatment on clinical manifestations in EAMG model. Weekly analysis of the global clinical score in EAMG mice (**A**) and area under the curve for the global clinical score (**B**). Analysis of CMAP following 10 stimulations (**C**), the specific maximal force (**D**) and relaxation time (**E**) in the tibialis anterior muscles of EAMG mice were performed at the killing time after 4 weeks of treatment. Data for EAMG (*n* > 12 per group) mice were obtained from 4 independent experiments. *P* values were obtained with ANOVA test. The *P* values are indicated as follows: * < 0.05; ** < 0.008; **** < 0.0001

In order to decipher whether a treatment that targets IL-23 may ameliorate MG disease, we investigated the effects of a monoclonal antibody anti-IL-23p19 on the classical EAMG mouse model (Fig. 1A–C). The EAMG mice receiving the anti-IL-23p19 treatment required 2 weeks to revert disease development (Fig. 1A). The area under the curve (AUC) of the disease kinetic displayed a significant decrease (AUC untreated = 21.23 versus treated = 14.05; *p 0.02* (Fig. 1B)).

To confirm that the decrease in the clinical score correlates with the level of integrity of the neuro-muscular-junction, we performed electromyography analyses on the tibialis anterior muscle at the end of the experiments. We measured the compound muscle action potential (CMAP) in response to nerve stimulation [30] to determine the excitability of the muscle fibers (the functionality of AChR clusters at the muscle membrane). The monoclonal anti-IL-23p19 antibody improved the decrease in CMAP (Fig. 1C) which is a specific feature observed in MG. Treatment also tended to ameliorate the maximal muscle force in EAMG mice (Fig. 1D) and increased tibialis anterior muscle relaxation time (Fig. 1E). These data suggest that blocking IL-23 may contribute to ameliorate MG manifestations.

### In the EAMG model, anti-IL-23 p19 treatment reduces the circulating level of IL-17 and anti-AChR IgG2b antibodies

To determine whether the effect of the anti-IL-23p19 treatment on EAMG clinical manifestations was related to IL-17 levels and the production of anti-AChR antibodies, we analyzed these factors in the mouse serum.

**Fig. 2:**
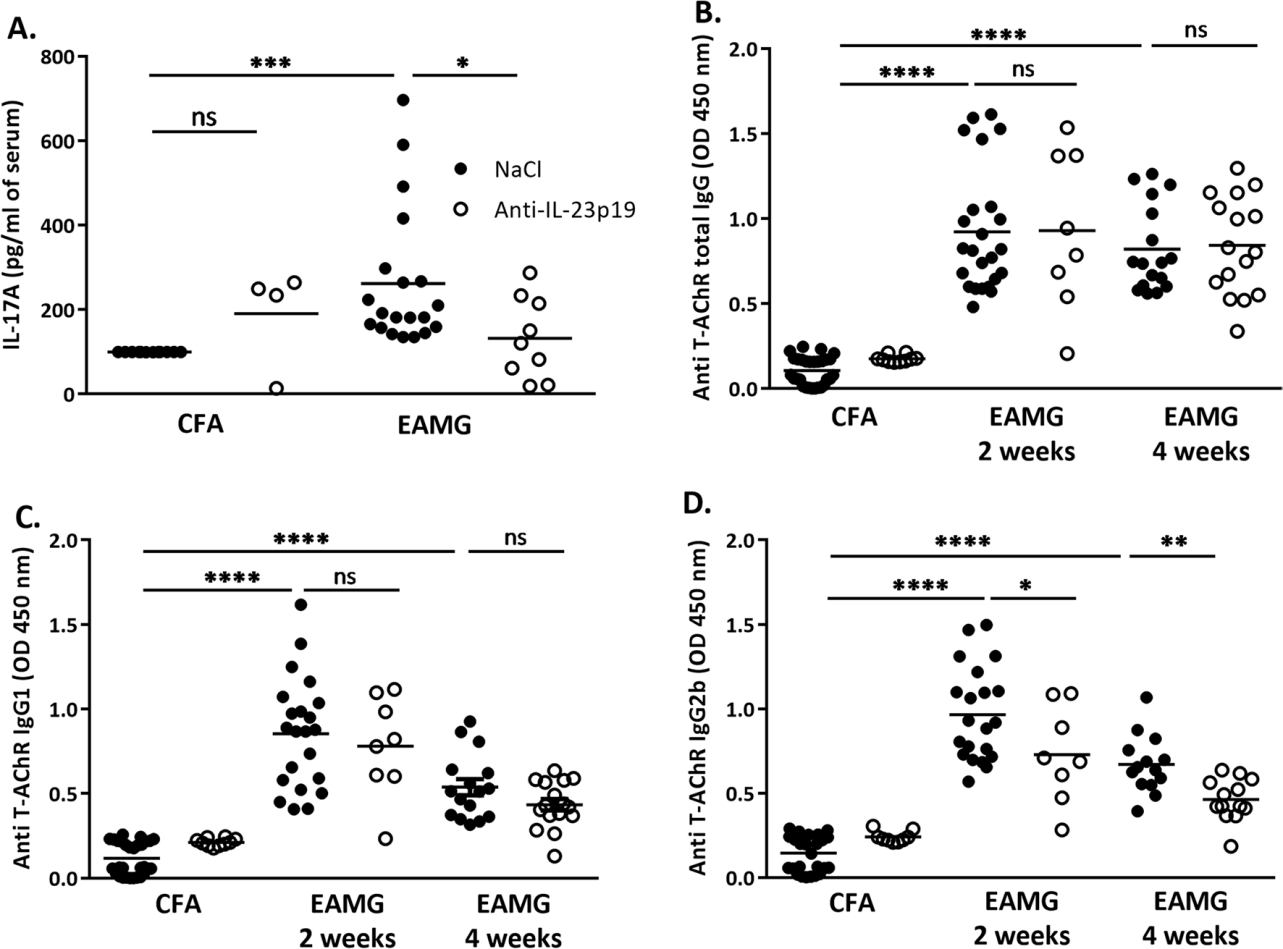
Effects of anti-IL-23p19 treatment on IL-17 and antibody levels in EAMG. ELISA analysis of the serum levels of IL-17 (4 weeks after treatment) (**A**), total anti-T-AChR antibodies (**B**), anti-T-AChR IgG1 subtype antibodies (**C**) and anti-T-AChR IgG2b subtype antibodies (**D**) in CFA and EAMG mice. The levels of anti-T-AChR antibodies of subtypes IgG1 and IgG2 were normalized to the total IgG levels. Analyses were performed in duplicate on serum obtained after 2 weeks or 4 weeks of treatment. For each treatment time, data were obtained from 2 independent experiments (EAMG mice n ≥ 8 per group). Each point represents an individual mouse. *P* values were obtained with an ANOVA test. The *P* values are indicated as follows: * < 0.05; **0.0015; ***0.0002; ****0.0001

First, and as previously reported in rat EAMG [32], we observed an increase in the IL-17 serum level of EAMG mice (Fig. 2A), an increase reversed by the anti-IL-23p19 treatment (Fig. 2A). Second, we observed that even though the levels of total anti-TAChR-IgGs and of anti-TAChR IgG1 isotype were not significantly modified by the anti-IL-23 treatment (Fig. 2B and C), the pathogenic anti-TAChR-IgG2b isotype was significantly reduced after 2 weeks of treatment (Fig. 2D). This decrease could explain the diminution of the clinical manifestations.

### Anti-IL-23p19 treatment stimulates molecules involved in the muscle regenerative and repair capacity

As the muscle is not a passive target of the anti-AChR antibody attack [33], we investigated the effects of the anti-IL-23p19 treatment on muscle-specific markers such as muscle regeneration process markers, previously shown to be altered in skeletal muscle of EAMG mice [33].

**Fig. 3:**
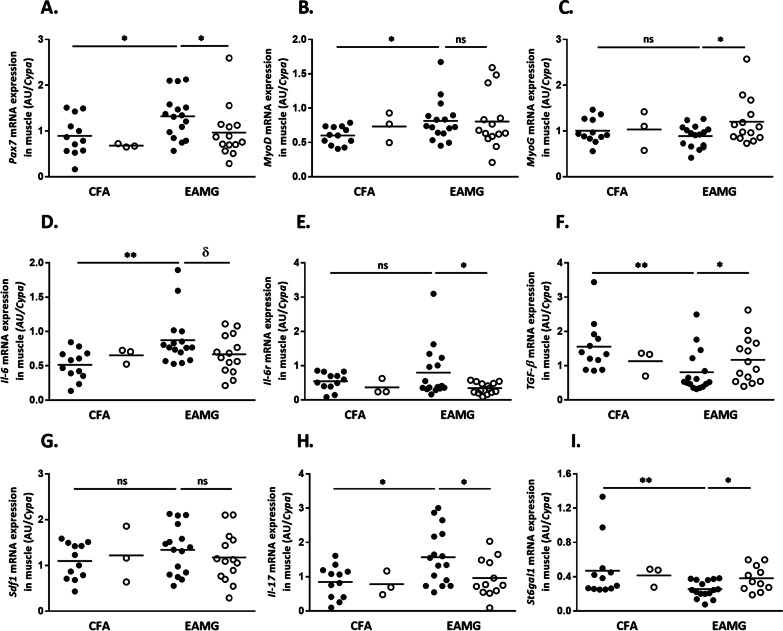
Effects of anti-IL-23p19 treatment on the activation of satellite cells and the inflammation status in EAMG muscle. mRNA expression analysis of *Pax7* (**A**), *MyoD* (**B**), *MyoG* (**C**), *Il-6 *(**D**), *Il-6r *(**E**), *Tgf-β* (**F**) Sdf1 (**G**) *Il-17 *(**H**)* and St6gal1 *(**I**) in tibialis anterior muscles from CFA and EAMG mice. Analyses were done after 4 weeks of treatment. Data are obtained from two representative experiments. There were *n* > 3 mice per group. Each point represents an individual mouse. mRNA expression was determined in duplicate by quantitative RT-PCR. mRNA levels are expressed as arbitrary unit (AU) normalized to *Cypa.* *P* values were obtained with a *t*-test. The *P* values are indicated as follows: δ = 0.0625; * < 0.05; ** < 0.003

We observed that the treatment had significant effects on the expression of functional markers of satellite cells (SC) after 2 weeks (Additional file 1: Fig. S1) that persisted after 4 weeks (Fig. 3). As shown in Fig. 3A, *Pax7* expression in non-treated EAMG mice was significantly higher than that in controls. Interestingly, EAMG mice treated with anti-IL-23p19 antibody showed a significant reduction in *Pax7* expression within the tibialis anterior muscle (Fig. 3A). After activation, SCs are known to differentiate into myoblasts expressing *Myo*D while a pool of SCs will go back to steady state [34]. Non-treated EAMG mice presented significantly increased expression of *MyoD*, which tended to be reverted by the treatment to the level of control mice after 2 but not at 4 weeks (Additional file 1: Figs. S1 and Fig. 3B). We also analyzed the expression of *MyoG*, a transcription factor and marker of myoblast maturation along the myogenic lineage [34]. Figure 3C shows that *MyoG* was overexpressed in the muscle of anti-IL-23 treated EAMG mice (Additional file 1: Figs. S1 and Fig. 3C). These data suggest that the treatment indirectly may contribute to orientate SCs to return to steady state, and to complete correctly the differentiation for the SCs that are already been engaged in muscle fiber regeneration/reparation process.

Various studies have shown that the muscle cytokine environment also plays major roles in cellular signaling and processes to influence SC function. For instance, IL-6 signaling is involved in the activation of SCs [35]. Therefore, we analyzed the expression of *Il-6* and its receptor, *Il-6r,* in skeletal muscle. We found that non-treated EAMG mice had a significantly increased expression of only *Il-6* compared with controls (Fig. 3D). Interestingly, anti-IL-23p19 treatment modulated the expression of *Il-6* and *Il-6r* (Fig. 3D and E), suggesting that the treatment impacts expression of cytokines involved in muscle SC activation. Classically, some activated SCs differentiate into myoblasts which fuse and form new myotubes or integrate the regenerating fibers. This process is promoted by numerous factors, including TGF-β1 [34] and SDF1 [36], and is altered by IL-17 [37]. We observed that the decreased muscle expression of *Tgf-β1* in EAMG mice was cancelled by anti-IL-23p19 treatment (Fig. 3F) while no change was observed for *Sdf1* expression (Fig. 3G). However, we observed a significant overexpression of *Il-17* in non-treated EAMG mice compared with controls. Interestingly, mice receiving the anti-IL-23 treatment showed a significant decrease in *Il-17* expression (Fig. 3H), even though the cells producing IL-17 in the muscle remained to be identified. These data suggest that the treatment modulated the expression of factors involved in SC fusion and maturation that could contribute to ameliorate the overall clinical status (Fig. 1A). Moreover, we also analyzed the mRNA expression of *ST6GAL1*, a membrane protein that catalyzes the transfer of sialic acid to galactose substrates in IgGs, decreasing their pathogenicity. We observed that *St6gal1* expression was significantly increased in the muscle following anti-IL-23p19 treatment (Fig. 3I).

Altogether, within the muscle, anti-IL-23 treatment seems to activate various mechanisms that lead to decrease the antibody-induced pathogenicity at the neuromuscular junction and to maintain muscle regenerative capacity. The detailed mechanism responsible for these effects remains to be further deciphered.

### In the MG-NSG model, the anti-IL-23p19 treatment improves MG manifestations

Whether the classical EAMG model provides evidence of significant amelioration of the muscle function by the anti-IL-23p19 treatment, this model is distinct from the human pathology as it provides no clue on the effects of such treatment on the thymus. We thus explored the effects of the treatment on a humanized mouse model in which mice were engrafted with human MG thymic fragments [27].

**Fig. 4:**
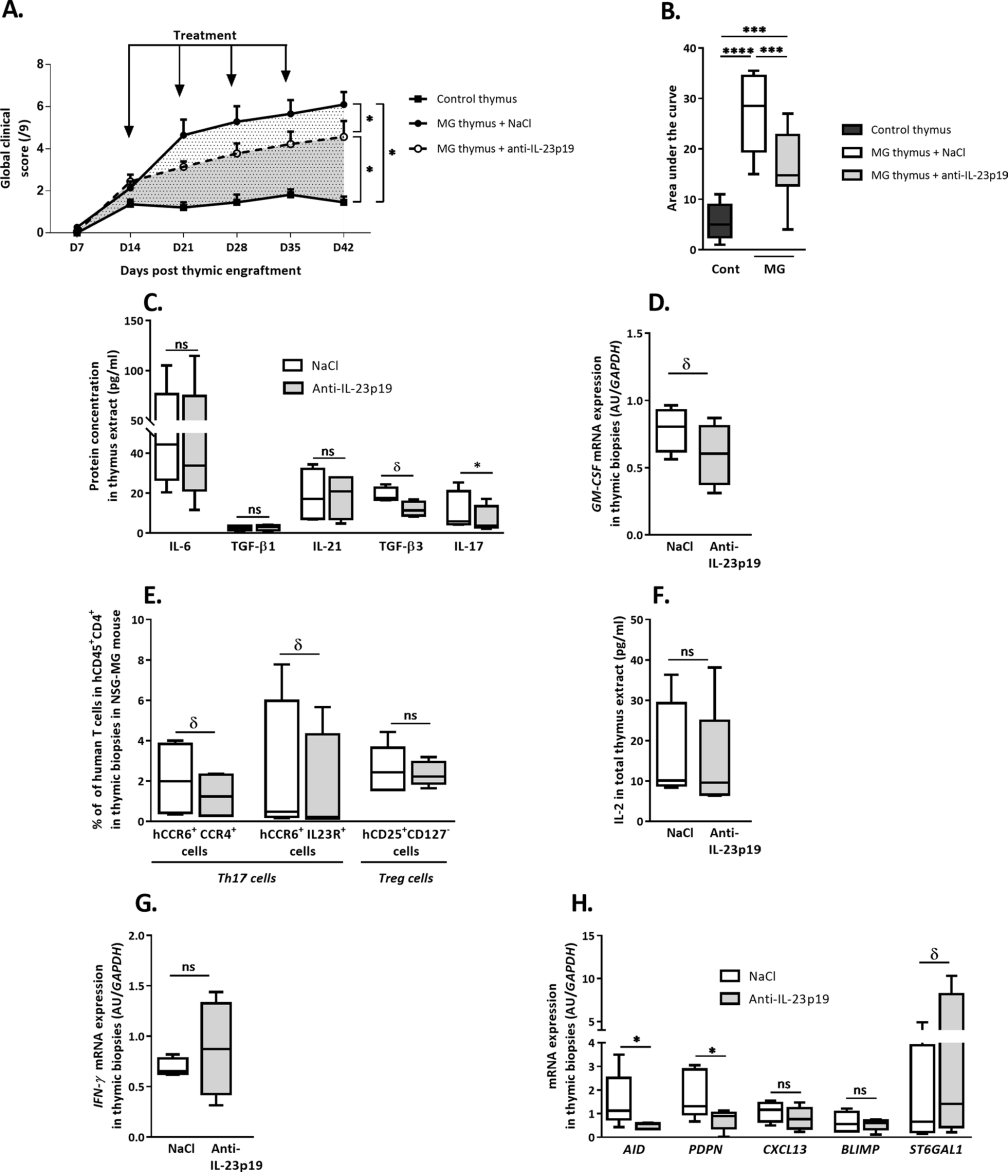
Effects of anti-IL-23p19 treatment in the NSG-MG model. Weekly analysis of the global clinical score in NSG-MG mouse model (**A**) and area under the curve for the global clinical score (**B**). Protein analyses of Th17-related cytokines (IL-6, TGF-β1, IL-21, TGF-β3 and IL-17A) (**C**) and mRNA expression of *GM-CSF* (**D**) in human AChR^+^ MG thymic biopsies engrafted in NSG-MG mice. Flow cytometry analyses of CCR6^+^CCR4^+^ T cells, CCR6^+^IL-23R^+^ T cells (Th17 cell subsets) and CD25^+^CD127^−^T cells among CD4^+^ single-positive T cells (**E**) in engrafted human thymic biopsies. Protein analysis of IL-2 (**F**) in human AChR^+^ MG thymic biopsies engrafted in NSG-MG mice. mRNA expression of *IFN-γ* in human thymic biopsies (**G**). mRNA expression levels of B cell markers (*AID, PODOPLANIN****,**** CXCL13****,**** BLIMP1* and *ST6GAL1)* (**H**) in AChR^+^ MG thymuses engrafted in NSG-MG mice. All analyses were performed at day 42 after thymic engraftment in NSG-MG mice treated for 4 weeks with saline solution (NaCl) or anti-IL-23p19 antibody. All the data are from at least 4 different experiments performed with thymic biopsies obtained from different MG patients. For each thymic biopsy, there were *n* > 3 mice per treatment condition. Each box represents the mean value of at least 4 experiments. Protein and mRNA analyses were performed, respectively, by ELISA and quantitative RT-PCR. mRNA expression results were normalized to *GAPDH* and are expressed as arbitrary unit (AU). *P* values were obtained using an Anova test (Graphs **A**, **B**) and Wilcoxon matched paired test (Graphs **C**–**H**). The *P* values are indicated as follows: *δ* = 0.0625; * < 0.05; ***0.0008; ****0.0001

We observed a significant amelioration of the clinical score of NSG-MG mice receiving the anti-human IL-23p19 demonstrating that the treatment was efficient on the MG manifestations in this alternative MG mouse model (Fig. 4A and B). Of note, the treatment also slightly decreased Th17 cell subsets in the circulation and spleen, while the decrease in the level of human anti-AChR antibodies was not significant in the NSG MG model (Additional file 1: Fig. S2).

### In the MG-NSG model, the anti-IL-23p19 treatment decreases the pathogenic Th17 signature in AChR^+^ MG thymuses

We then investigated whether the IL-23p19 treatment had a deleterious effect on the general physiology of the engrafted human thymic biopsies. Our data demonstrated that the treatment did not alter the vascularization of the engrafted human thymic fragments, neither physiological thymic characteristics (i.e., thymic histological structures (K5/K14^+^ cells) or major thymic cell populations) or cell export function and regulation (Additional file 1: Fig. S3).

The treatment induced no changes in the engrafted thymus levels of IL-6 and TGF-β1, which are cytokines involved in the initial development of Th17 cells (Fig. 4C), or IL-21, a cytokine required for differentiation and regenerative feedback mechanism of Th17 cells (Fig. 4C). However, we observed changes in the Th17 cell pathogenic signature. TGF-β3, a cytokine-induced by IL-23 and involved in the development of pathogenic Th17 cells [16], showed decreased thymic expression in mice treated with the anti-IL-23p19 antibody (Fig. 4C). A similar result was obtained for the thymic level of IL-17A, the classical cytokine produced by Th17 cells (Fig. 4C). In addition, the mRNA expression of GM-CSF, a cytokine critical for pro-inflammatory Th17 cells, displayed a decrease in the MG thymuses following treatment (Fig. 4D).

Of note, in the classical EAMG model, the treatment induced similar effect in the spleen after 4 weeks of treatment (Additional file 1: Figs. S4 and S5). The mRNA expression levels of cytokines involved in the initial development of Th17 cells, such as *Il-6* (Additional file 1: Fig. S4A) and *Tgf-β1* (Additional file 1: Fig. S5A). By contrast, the treatment tends to decrease the mRNA expression of *Tgf-β3*, *Il-17a*, *Il-22* (Additional file 1: Fig. S5 B–D), Th17 cell pathogenic signature, without significant effects on *Il-10* mRNA expression (Additional file 1: Fig. S5E).

These observations were corroborated by the reduction in the percentages of two subpopulations of human Th17 cells, CD4^+^CCR6^+^CCR4^+^ T cells and pathogenic CD4^+^CCR6^+^IL-23R^+^ T cells, observed within the engrafted thymuses (Fig. 4E).

We also assessed the treatment impact on the regulatory T (Treg) cell signature. We observed no changes in the thymic percentage of Treg cells (defined by the CD4^+^CD25^+^CD127^−^ phenotype) (Fig. 4E) or in the thymic IL-2 protein level (Fig. 4F). In addition, the expression of IFN-γ was unchanged (Fig. 4G). Altogether, these data show that IL-23-targeted treatment affected the MG thymus fragments by decreasing pathogenic Th17 cell and inflammatory markers without altering the Treg cells, likely restoring the equilibrium between Th17 and Treg cells.

### In the MG-NSG model, anti-IL-23p19 treatment modulates thymic expression of molecules involved in antibody production

We have previously demonstrated that AChR^+^ MG thymuses display increased expression of activation-induced cytidine deaminase (AID) (a protein involved in B cell somatic hypermutation) and PODOPLANIN (a protein stabilizing eGCs) [17]. We wondered whether the decrease in the Th17 signature might also impact eGCs and their related gene regulators. The expression of *AID* and *PODOPLANIN* mRNA in MG-engrafted thymuses showed a decrease in both markers in the treated group of NSG-MG mice (Fig. 4H). Of note, in the classical EAMG model, the treatment induced similar effect in the spleen after 4 weeks of treatment (Additional file 1: Fig. S4B, C).

However, the treatment did not affect the thymic expression of the B-cell chemoattractant *CXCL13* (Fig. 4H) or B-lymphocyte induced maturation protein-1 (*BLIMP1*) (Fig. 4H). The *BLIMP1* gene is an IL-21 target [38], and the BLIMP1 protein is involved in B-cell maturation and activation.

*ST6GAL1* is known to be regulated by the Th17/IL-23 pathway [19]. We observed a slight increase in the thymic expression of *ST6GAL1*, although the increase was not significant (Fig. 4H). Altogether, these data suggest that the anti-IL-23p19 treatment did not reduce the chemoattraction of B cells into the thymus but decreased their activation, preventing their organization in eGCs within the thymus.

## Discussion

Th17 cells are known for their implication as drivers of autoimmunity. IL-23 promotes the development of autoimmune diseases such as multiple sclerosis, rheumatoid arthritis, and systemic lupus erythematosus by stimulating pathogenic Th17 cells and autoantibody production. Therefore, IL-23 is a potential target for modulating autoimmune responses and pathogenic Th17 cell effects. Ongoing clinical trials tend to demonstrate the beneficial effects of blocking the IL-23/Th17 pathway in inflammatory pathologies. AChR^+^ MG is a complex autoimmune disease that affects thymus physiology and reduces muscle function. AChR^+^ MG is also characterized by the over-activation of the IL-23/Th17 pathway [17]. Here, we used two complementary MG mouse models, and demonstrated that an anti-IL-23p19 treatment significantly alleviated the clinical manifestations in both MG models. By studying the molecular mechanisms involved, we showed that inhibiting the IL-23 pathway had a beneficial effect on all the tissues involved in MG pathology.

### Beneficial effects of the treatment on the global Th17/IL-17 signature in MG

We have previously provided evidence that in the AChR^+^ MG thymus, there is a Th17 cell signature characterized by the presence of effector pathogenic Th17 cells that cross-talk with TECs to over-produce IL-17. Treg cells partially enhance the inflammation by secreting IL-17 and IL-21, Th17 cytokine signatures [15]. In addition to the thymic Th17 cell signature, various studies have shown that MG patients present higher levels of circulating IL-17 compared to healthy controls [39, 40]. In peripheral blood mononuclear cells from MG patients, activated Th1/Th17 autoreactive T-cells also produce pathogenic cytokines, including IL-17, IFN-γ and GM-CSF [41]. In fact, elevated IL-23 levels are found in AChR^+^ MG thymuses and in peripheral blood, emphasizing the activated pathogenic Th17 cell pathway in different body compartments [17]. However, one study did not observe a difference in the IL-17 blood level in MG patients compared with healthy controls, but differences in ethnicity, sex, age, treatments (for example, an effect of immunosuppressants) or methodology (for example, some experiments require activation of peripheral blood mononuclear cells with anti-CD3 antibodies) may account for this discrepancy [42].

Animal models of MG also recapitulate the Th17/IL-17 signature observed in the human pathology. Sudres et al. showed that human MG thymus fragments are transposable and maintained in the humanized mouse model of AChR^+^ MG, including overexpression of IL-17, IL-6, TNF-α, and IFN-γ [27]. The classical MG mouse model has also provided evidence of the active role of the Th17/IL-17 pathway. Hence, mice deficient in IL-17 display a moderate experimental autoimmune MG disease severity [26]. In addition, the use of an anti-IL-6 antibody induces a down-regulation of the global Th17 signature without affecting Treg cells and suppresses EAMG in rats [32].

As mentioned before, two steps are required to achieve the differentiation of pathogenic Th17 cells. The first step, involving IL-6 and TGF-β1, gives rise to non-pathogenic Th17 cells that became pathogenic (CD4^+^CCR6^+^IL-23R^+^ cells) in the presence of IL-23 and TGF-β3 [16]. In our study, we have chosen to target the pathogenic Th17 cells with an anti-IL-23p19 treatment. We clearly demonstrated an improvement of MG manifestations in both mouse models. The impact of the treatment on pathogenic Th17 cells was illustrated by a decrease in IL-17 serum levels and even in muscle in EAMG mice, and a decrease in pathogenic Th17 cells and of IL-17 in engrafted thymuses. The anti-IL-23p19 antibody altered the thymus, muscle, and circulating levels of IL-17 without modifying cytokines required for the development of Th1 cells (IFN-γ) and Treg cells (IL-2 and TGF-β1). Therefore, the anti-IL-23p19 antibody may reduce pathogenic Th17 cells, restore the equilibrium between inflammatory Th17 and Treg cells and consequently reduce Th17 cell-related inflammation in AChR^+^ MG in the thymus as well as in the periphery. This agrees with studies showing that anti-IL-23p19 antibody may inhibit disease progression in other experimental autoimmune diseases by reducing pathogenic Th17 cells and their related cytokines [43].

### Targeting IL-23 affects eGC development and autoantibody production

GCs are the site of B cell selection and maturation in secondary lymphoid organs in response to infection and immunization. In inflammatory conditions, eGCs can also develop in non-lymphoid organs in cancers or autoimmune diseases. CD4^+^ T cells interact with GC B cells to initiate the GC reaction and to support its stability by co-stimulation and cytokine signals, a phase that is quickly followed by the help of T follicular helper (Tfh) cells. In GC, B cells cross-react with Tfh cells producing IL-21, IL-4, and pathogenic cytokines such as IL-17 and IFN-γ [44]. IL-17 signaling contributes significantly to the development and survival of the autoantibody producing B cells [44]. Whether IL-23 is not essential for GC formation, IL-23 is important for B cell class-switching recombination and promotes the induction of DNA excision repair genes in GC B cells [45]. In humans, Tfh generation relies on TGF-β, IL-12, and IL-23, while in mice, it depends on IL-6, IL-21, and Bcl6 [46]. Animal models have demonstrated the important role of IL-17 in GC development. In the BXD2 mouse model, which spontaneously develops erosive arthritis and glomerulonephritis in the presence of autoantibodies, IL-17 plays a critical role in developing GCs and activating B cells by inducing AID expression and somatic hypermutation in B cells [47]. Indeed, in the EAE mouse model, Th17 cells co-expressing IL-17^+^ and podoplanin have been located in eGCs and blocking podoplanin reduces the number of eGCs [18].

In early-onset AChR^+^ MG patients, the thymus is characterized by eGCs with B cells producing anti-AChR antibodies [1, 8, 27]. AChR^+^ MG thymuses overexpress molecules playing a central role in eGC development, such as IL-17, IL-23, IL-21, IFN-γ, TGF-β1/3, IL-6 [1, 8, 27]. At the same time, in the EAMG mouse model, a deficiency of IL-6 is reported to induce a significant decreased in size and number of GCs in the spleen [48]. Here, we showed that blocking IL-23p19 in the humanized MG mouse model and in the classical EAMG model, decreases the expression of signaling molecules involved in GC homeostasis, including IL-17, AID and podoplanin in engrafted human MG thymuses fragments and in spleens.

In addition, whether anti-IL-23p19 may have disturbed GC stability or size, we also obtained a diminished antibody production for the IgG2b subtype, which is the pathogenic IgG subtype in mouse, without affecting other IgG subtypes in the classical MG mouse model, while no change in total human antibody titer was observed in the humanized MG model. This suggests that IL-23 activation may play a role in the IgG2b subtype antibody production in MG thymus by modulating class-switching recombination actors and IgG2b related genes. Interestingly, in the BXD2 mouse invalidated for IL-23p19, it is reported a decreased development only for IgG2b producing B-cells [45], a result that emphasizes a previous study reporting that high IL-17 titer enhances IgG2b but not IgG1 CSR [49].

Therefore, the decreased disease outcome observed in the two mice models may not only rely on the reduced total production of pathogenic autoantibodies, but may result in other disturbances occurring in the germinal centers and/or in the periphery. Indeed, recently, Jiang et al. have demonstrated that the AChR^+^ MG thymus is the source and reservoir of plasma cells secreting AChR autoantibodies that may be exported to the periphery and remain active and deleterious for the muscle after thymectomy [9]. We have observed that anti-IL-23p19 treatment stimulates an increased expression of ST6GAL1 in the two MG models and in different tissues. ST6GAL1 protein controls the sialylation-dependent anti-inflammatory function of IgG [50]. Thus, we hypothesized that even though the total IgG titer may remain unchanged, their IgG sialylation status may have been increased, leading to less pathogenic antibodies.

### Potential impact of the treatment on MG muscle

As we clearly demonstrated an improvement of MG manifestations in both mouse models, we investigated whether the decrease in the Th17-related inflammation has also an impact on muscle homeostasis and function.

Our results show that EAMG muscles present an increased expression of IL-17 that can be reversed by the anti-IL-23p19 treatment. Muscle is sensitive to inflammation. In non-pathological conditions, IL-17 and IL-23 promote neutrophil activation and muscle damage following prolonged endurance exercise [51]. In vitro, IL-17 alone or in conjunction with other cytokines (i.e., TNF-α and IL-1β) induces myokine secretion (IL-6 and IL-8) by myocytes [52]. Even though the cells producing IL-17 in EAMG muscle remain to be identified, the myasthenic muscle may already be subject to physiological and functional defects that were potentially lowered by the anti-IL-23p19 treatment. Hence, our results from EAMG muscle (tibialis anterior) demonstrated that by controlling Th17 cell development and IL-17 secretion, we have probably induced a combined beneficial effect on muscle function through reduced production of anti-AChR antibodies (IgG2b subtype) and modulation of myokine secretion.

Attia et al. showed that anti-AChR antibodies activate satellite cells in AChR^+^ MG patients and in the EAMG mouse model [33]. SCs develop upon stimulation and present differential expressions of transcription factors throughout their differentiation into myotube. For instance, after activation, SCs express Pax7 and MyoD and begin to proliferate. Then, SCs downregulate the expression of Pax7 and MyoD and upregulate MyoG, allowing progenitors fusion and differentiation into myotubes [34]. However, in myasthenic muscles (mouse and human), the regeneration capacity is functionally altered, reflected by impairment of the fusion and maturation of newly formed fibers during muscle regeneration [33]. Anti-IL-23p19 induces upregulation of MyoG. Therefore, our data demonstrate that by controlling the IL-23/IL-17 pathway, we can also modulate the expression of markers involved in the activation/differentiation of SCs. The treatment tends to promote the differentiation of SCs into myotubes in EAMG muscle that consequently ameliorates the muscle regeneration capacity. This hypothesis is compatible with the data by Kocic et al., suggesting a potentially active role of IL-17 in muscle fiber differentiation [37].

Interestingly, Der Vartanian et al. showed that the efficient activation of the muscle regenerative pathway (Notch signaling pathway) requires *N*-glycan modifications on the cell surface [53]. In mice deficient for St6gal1, a decrease in α2, 6 sialylation of *N*‐glycans favors the differentiation of most Pax7^+^ cells and provokes a significant loss of reserve cells [21]. Our data show that by decreasing Th17-related inflammation in EAMG muscle, we also modulate the muscle expression of St6gal1, which may enhance *N*-glycan transduction pathways involved in myotube differentiation.

## Conclusion

We and others have demonstrated that Th17 cells have a critical role in the development of AChR^+^ MG in the thymus and muscle. We showed that a treatment that targets the pathogenic Th17, with an anti-IL-23p19 monoclonal antibody, is effective in decreasing inflammation in the MG thymus and controlling the formation of eGCs, which are a hallmark of AChR^+^ MG thymuses and a source of autoantibodies. The treatment ameliorates clinical symptoms by reducing autoantibodies and improving muscle physiology. Here, we showed for the first time the ability of an anti-IL-23p19 monoclonal antibody to reverse and ameliorate the physiopathology event occurring in AChR^+^ MG. More, monoclonal antibodies that target the IL-23/Th17 cell pathway are emerging as therapeutic tools to treat autoimmune diseases. For instance, ustekinumab, an anti-IL23p40 monoclonal antibody, is now used in the treatment of psoriasis and Crohn’s disease. Guselkumab, an anti-IL-23p19 monoclonal antibody, is now approved to treat psoriasis. A clinical study (based on medicine repositioning) that aims to investigate the potential effects of such therapeutic option for early-onset AChR^+^ MG patients should be envisaged.

## Supplementary Information

**Additional file 1: Figure S1.** Anti-IL-23p19 treatment decreases the activation of SCs and inflammation in EAMG muscle after 2 weeks of treatment. mRNA expression of *Pax7 ****(*A)**, *MyoD* **(B)**, *MyoG* **(C)**, Il-6 **(D)**, *Il-6r* **(E)**, *Tgf-β* **(F)** and *Il-17a* **(G)** in the Tibialis anterior muscle of CFA and EAMG mice treated with or without anti-IL-23p19 antibody. mRNA analyses were performed in duplicate after 2 weeks treatment. Data were obtained from 2 independent experiments. There were *n* > 4 mice per group. Each point represents an individual mouse. mRNA expression were determined in duplicate by quantitative RT-PCR. mRNA levels are expressed as arbitrary unit (AU) and normalized to *Cypa.* *P* values were obtained with a t-test. *P* value are *δ* = 0.06 to 0.05; * < 0.05;**0.005; *** 0.0007. **Figure S2.** Effect of anti-IL-23p19 treatment on human Th17/Treg cells and antibodies in the spleen and blood of engrafted NSG mice. Analysis by flow cytometry of human T cells in the spleens of NSG-MG mice **(A)**. ELISA analysis of human anti-AChR antibodies in the blood of NSG-MG mice 28 days after thymic engraftment **(B)**. Flow cytometry analyses were performed at day 42 after thymic engraftment in NSG-MG mice treated with saline solution (NaCl) or anti-IL-23p19 antibody. Each point represents the mean value per experiment for each thymic biopsy obtained from one donor. Each point is from at least 4 mice. All data are from at least 4 different experiments done with thymic biopsies obtained from different AChR^+^ MG patients. *P* values were obtained with Wilcoxon matched paired test. **Figure S3.** Anti-IL-23p19 treatment does not induce global physiological changes in the NSG-MG mouse model. mRNA expression levels of Keratin 14 **(A)** in AChR^+^ MG thymuses engrafted in NSG-MG mice. Representative images of vascularized human MG thymuses after engraftment in mice without **(B)** or with treatment **(C)**. Flow cytometry analyses of human T cells in engrafted human MG thymuses **(D)**, in the blood **(E)** and in spleens **(F)** of NSG mice. ELISA quantification of total human immunoglobulins in the serum of NSG-MG mice **(G)**. Images were acquired with a Zeiss Axio Observer Z1 inverted microscope using 20× magnification. In the flow cytometry graphs, each point represents the mean of the percentage of cells for an experiment. All analyses were performed at day 42 after engraftment in NSG-MG mice treated with saline solution (NaCl) or anti-IL-23p19 antibody. The data are from at least 3 different experiments performed with at least 3 thymic biopsies obtained from different MG patients. For each thymic biopsy, there were *n* > 3 mice per treatment condition. *P* values were obtained using the Wilcoxon matched paired test (**A–F**) and an ANOVA test (**G**). **Figure S4.** Anti-IL-23p19 tends to reduce markers of eGCs in the spleens of EAMG mice. mRNA expression of Il-6 (**A)**, *Podoplanin* **(B)** and *Aid* **(C)** in the spleens of CFA and EAMG mice treated with or without anti-IL-23p19 antibody. mRNA analyses were performed in duplicate after 2 weeks or 4 weeks of treatment by quantitative RT-PCR. For each treatment time, data were obtained from 2 independent experiments. *n* > 4 mice per group. Each point represents an individual mouse. The mRNA results are expressed as arbitrary unit (AU) and normalized to *Gapdh*. *P* values were obtained with an ANOVA test. *P* value are as follows * < 0.05;**0.004; ****0.0001. **Figure S5.** Anti-IL-23p19 modifies markers of pathogenic Th17 cells in the spleens of EAMG mice. mRNA expression of *Tgf-β1* (**A)**, *Tgf-β3* **(B)**, *Il-17a* **(C)**, *Il-22* **(D)**, *Il-10* **(E)** in the spleens of CFA and EAMG mice treated with or without anti-IL-23p19 antibody. mRNA analyses were performed in duplicate after 2 weeks or 4 weeks of treatment by quantitative RT-PCR. For each treatment time, data were obtained from 2 independent experiments. *n* > 4 mice per group. Each point represents an individual mouse. The mRNA results are expressed as arbitrary unit (AU) and normalized to *Gapdh*. *P* values were obtained with an ANOVA test (δ = 0.05; * < 0.05;**0.007; ***0.001; ****0.0001).

## Data Availability

The datasets analyzed during the current study are available from the corresponding author on reasonable request.

## Abbreviations

AChR: Acetylcholine receptor
AID: Activation-induced cytidine deaminase
ANOVA: Analysis of variance
AUC: Area under the curve
BLIMP1: B-lymphocyte induced maturation protein-1
CFA: Complete Freund’s adjuvant
CMAP: Compound muscle action potential
EAMG: Experimental autoimmune myasthenia gravis
eGC: Ectopic germinal center
IFN-β: Interferon β
IgG: Immunoglobulin
IL-12: Interleukin 12
IL-17: Interleukin 17
IL-1β: Interleukin 1 beta
IL-21: Interleukin 21
IL-23: Interleukin 23
IL-6: Interleukin 6
MG: Myasthenia gravis
mTEC: Medullary thymic epithelial cell
*Myo*D: Myoblast determination protein 1
MyoG: Myogenin
NSG-MG: NSG humanized myasthenia gravis
Pax7: Paired Box 7
SC: Satellite cells
ST6gal1: β-Galactoside α 2,6-sialyltransferase 1
T-AChR: Torpedo acetylcholine receptor
TGF-β: Transforming growth factor beta
Th17: T helper cell subset 17
TNF-α: Tumor necrosis factor alpha
