# Supplementary material for: Blocking interleukin-23 ameliorates neuromuscular and thymic defects in myasthenia gravis
Source: J Neuroinflammation. 2023 Jan 13;20:9. doi: 10.1186/s12974-023-02691-3 (PMC9837970; doi:10.1186/s12974-023-02691-3)
Supplement: Supplementary file 1 — Additional file 1: Figure S1. Anti-IL-23p19 treatment decreases the activation of SCs and inflammation in EAMG muscle after 2 weeks of treatment. mRNA expression of Pax7 (A), MyoD (B), MyoG (C), Il-6 (D), Il-6r (E), Tgf-β (F) and Il-17a (G) in the Tibialis anterior muscle of CFA and EAMG mice treated with or without anti-IL-23p19 antibody. mRNA analyses were performed in duplicate after 2 weeks treatment. Data were obtained from 2 independent experiments. There were n > 4 mice per group. Each point represents an individual mouse. mRNA expression were determined in duplicate by quantitative RT-PCR. mRNA levels are expressed as arbitrary unit (AU) and normalized to Cypa. P values were obtained with a t-test. P value are δ = 0.06 to 0.05; * < 0.05;**0.005; *** 0.0007. Figure S2. Effect of anti-IL-23p19 treatment on human Th17/Treg cells and antibodies in the spleen and blood of engrafted NSG mice. Analysis by flow cytometry of human T cells in the spleens of NSG-MG mice (A). ELISA analysis of human anti-AChR antibodies in the blood of NSG-MG mice 28 days after thymic engraftment (B). Flow cytometry analyses were performed at day 42 after thymic engraftment in NSG-MG mice treated with saline solution (NaCl) or anti-IL-23p19 antibody. Each point represents the mean value per experiment for each thymic biopsy obtained from one donor. Each point is from at least 4 mice. All data are from at least 4 different experiments done with thymic biopsies obtained from different AChR+ MG patients. P values were obtained with Wilcoxon matched paired test. Figure S3. Anti-IL-23p19 treatment does not induce global physiological changes in the NSG-MG mouse model. mRNA expression levels of Keratin 14 (A) in AChR+ MG thymuses engrafted in NSG-MG mice. Representative images of vascularized human MG thymuses after engraftment in mice without (B) or with treatment (C). Flow cytometry analyses of human T cells in engrafted human MG thymuses (D), in the blood (E) and in spleens (F) of NSG mice [file 12974_2023_2691_MOESM1_ESM.docx]

# Blocking Interleukin-23 ameliorates neuromuscular and thymic defects in myasthenia gravis

**José A. Villegas^1^, Jérôme Van Wassenhove^1^, Judith Merrheim^1^,**

**Karen Matta^1^, Samy Hamadache^1^, Clémence Flaugère^1^, Pauline Pothin^1^, Frédérique Truffault^1^, Sébastien Hascoët^2^,**

**Nicola Santelmo^3^, Marco Alifano^4^, Sonia Berrih-Aknin^1^,**

**Rozen le Panse^1^ and Nadine Dragin^1*^**

^1^ Sorbonne Université, Inserm, Institut de Myologie, Centre de Recherche en Myologie, 75013 Paris, France

^2^ Hôpital Marie Lannelongue, Le Plessis-Robinson, France

^3^ Chirurgie Thoracique des deux Rives, Rhéna - Clinique de Strasbourg, Strasbourg, France

^4^ Department of Pathology, Cochin University Hospital Group, AP-HP, Paris-Descartes University, Paris, France

^*^ Corresponding author

Correspondence and requests for materials should be addressed to:

Dr Nadine DRAGIN, Sorbonne Université, Inserm, Institut de Myologie, Centre de Recherche en Myologie, 105 Bd de l’hôpital, 75013 Paris, France, Tel: 00 33 (0)1 40 77 81 27, Fax: 00 33 (0)1 40 77 81 29; nadine.dragin-mamavi@upmc.fr


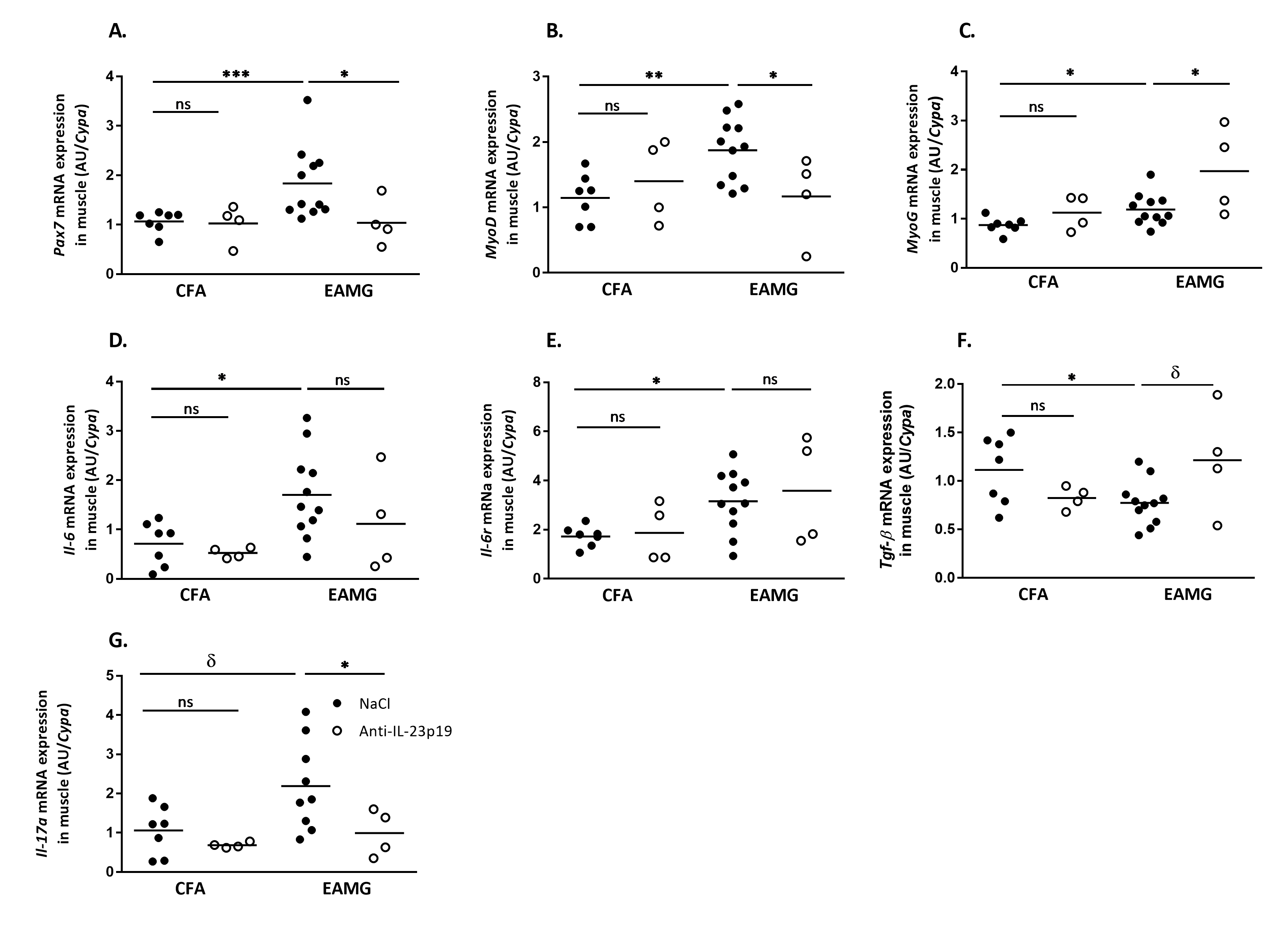


**Figure S1. Anti-IL-23p19 treatment decreases the activation of SCs and inflammation in EAMG muscle after 2 weeks of treatment.**

mRNA expression of *Pax7* ***(*A)**, *MyoD* **(B)**, *MyoG* **(C)**, Il-6 **(D)**, *Il-6r* **(E)**, *Tgf-β* **(F)** and *Il-17a* **(G)** in the Tibialis anterior muscle of CFA and EAMG mice treated with or without anti-IL-23p19 antibody. mRNA analyses were performed in duplicate after 2 weeks treatment. Data were obtained from 2 independent experiments. There were n>4 mice per group. Each point represents an individual mouse. mRNA expression were determined in duplicate by quantitative RT-PCR. mRNA levels are expressed as arbitrary unit (AU) and normalized to *Cypa.* P values were obtained with a t-test. P value are δ= 0.06 to 0.05; *<0.05;**0.005; *** 0.0007.


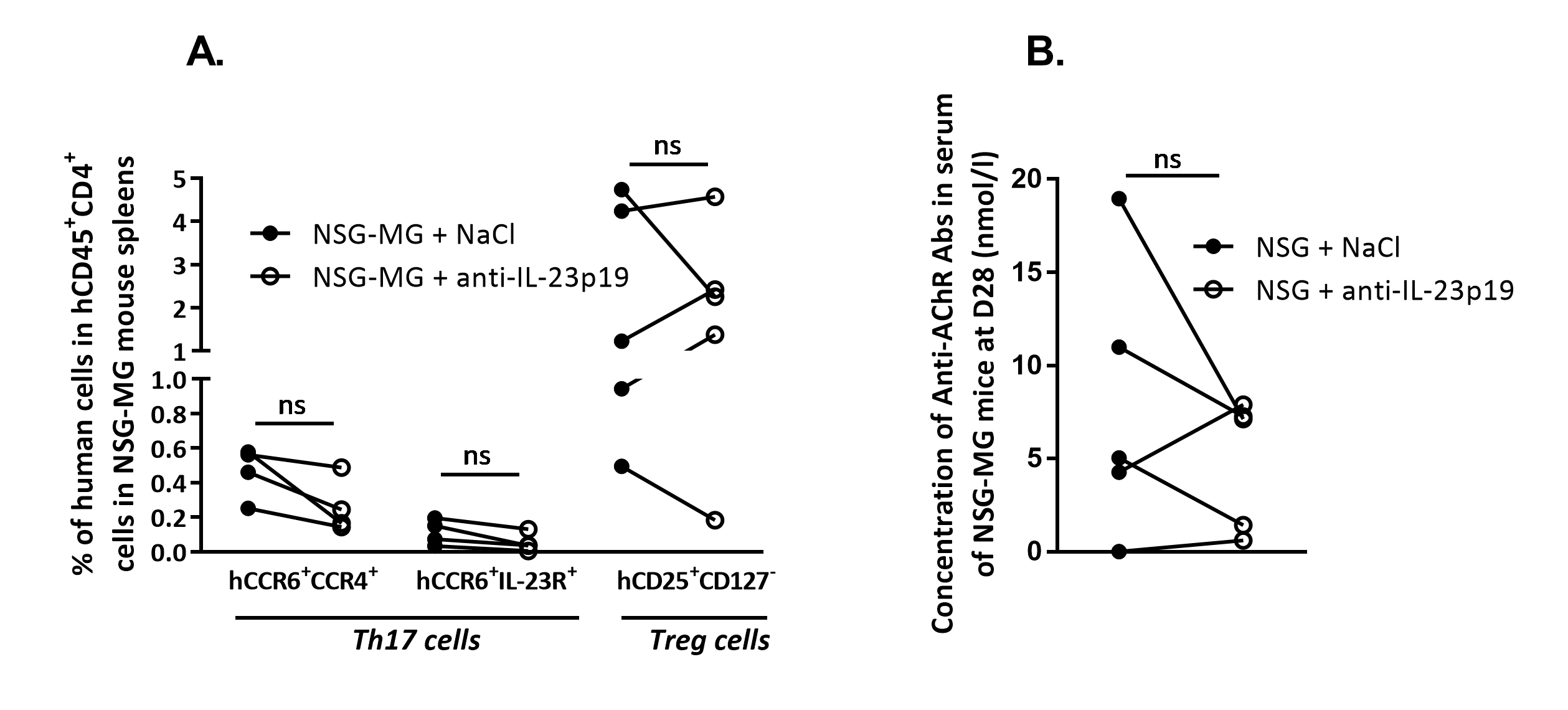


**Figure S2. Effect of anti-IL-23p19 treatment on human Th17/Treg cells and antibodies in the spleen and blood of engrafted NSG mice.**

Analysis by flow cytometry of human T cells in the spleens of NSG-MG mice **(A)**. ELISA analysis of human anti-AChR antibodies in the blood of NSG-MG mice 28 days after thymic engraftment **(B)**. Flow cytometry analyses were performed at day 42 after thymic engraftment in NSG-MG mice treated with saline solution (NaCl) or anti-IL-23p19 antibody. Each point represents the mean value per experiment for each thymic biopsy obtained from one donor. Each point is from at least 4 mice. All data are from at least 4 different experiments done with thymic biopsies obtained from different AChR^+^ MG patients. P values were obtained with Wilcoxon matched paired test.


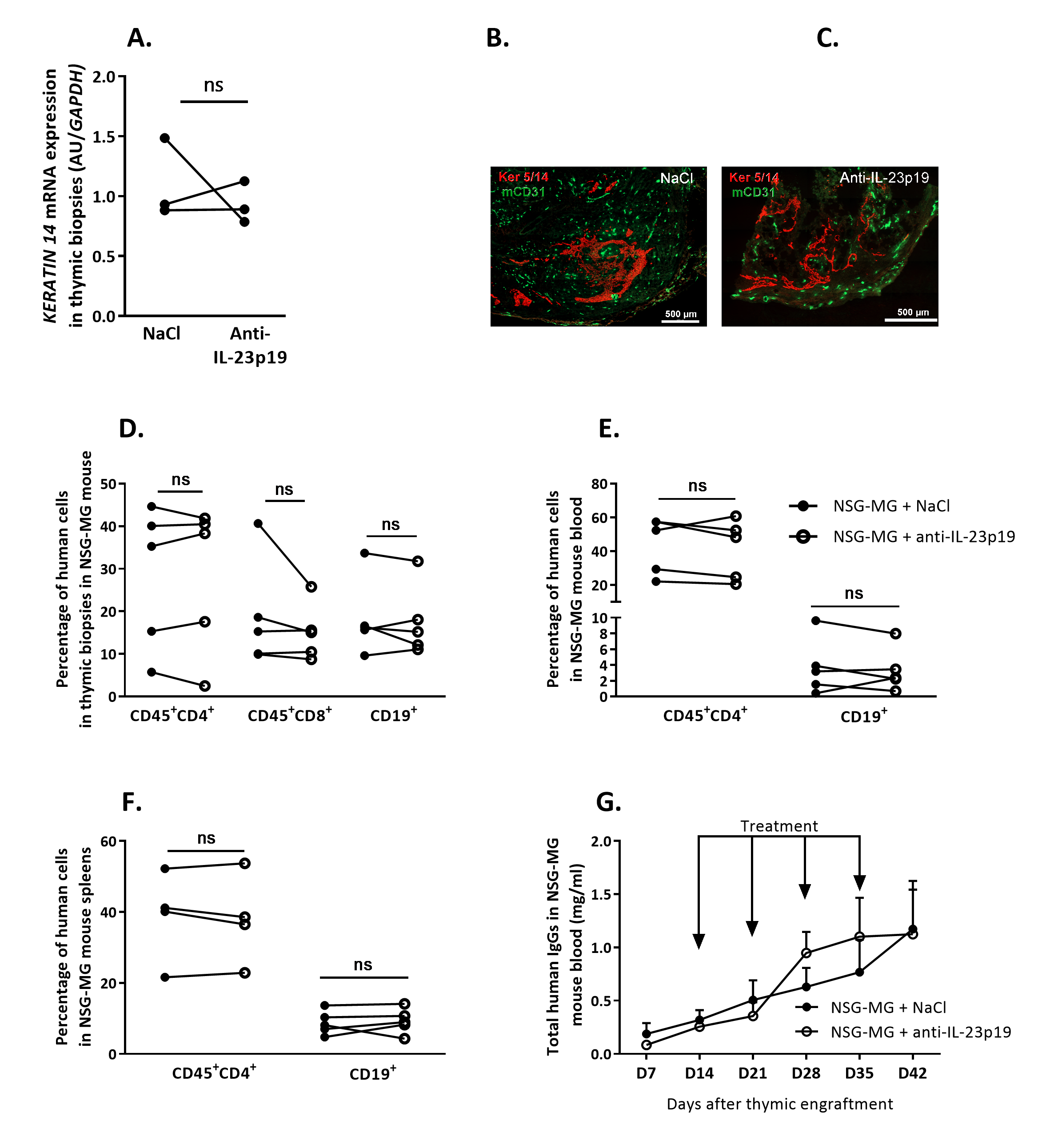


**Figure S3. Anti-IL-23p19 treatment does not induce global physiological changes in the NSG-MG mouse model.**

mRNA expression levels of Keratin 14 **(A)** in AChR^+^ MG thymuses engrafted in NSG-MG mice. Representative images of vascularized human MG thymuses after engraftment in mice without **(B)** or with treatment **(C)**. Flow cytometry analyses of human T cells in engrafted human MG thymuses **(D),** in the blood **(E)** and in spleens **(F)** of NSG mice. ELISA quantification of total human immunoglobulins in the serum of NSG-MG mice **(G)**. Images were acquired with a Zeiss Axio Observer Z1 inverted microscope using 20× magnification. In the flow cytometry graphes, each point represents the mean of the percentage of cells for an experiment. All analyses were performed at day 42 after engraftment in NSG-MG mice treated with saline solution (NaCl) or anti-IL-23p19 antibody. The data are from at least 3 different experiments performed with at least 3 thymic biopsies obtained from different MG patients. For each thymic biopsy, there were n>3 mice per treatment condition. P values were obtained using the Wilcoxon matched paired test (**A-F**) and an ANOVA test (**G**).

**
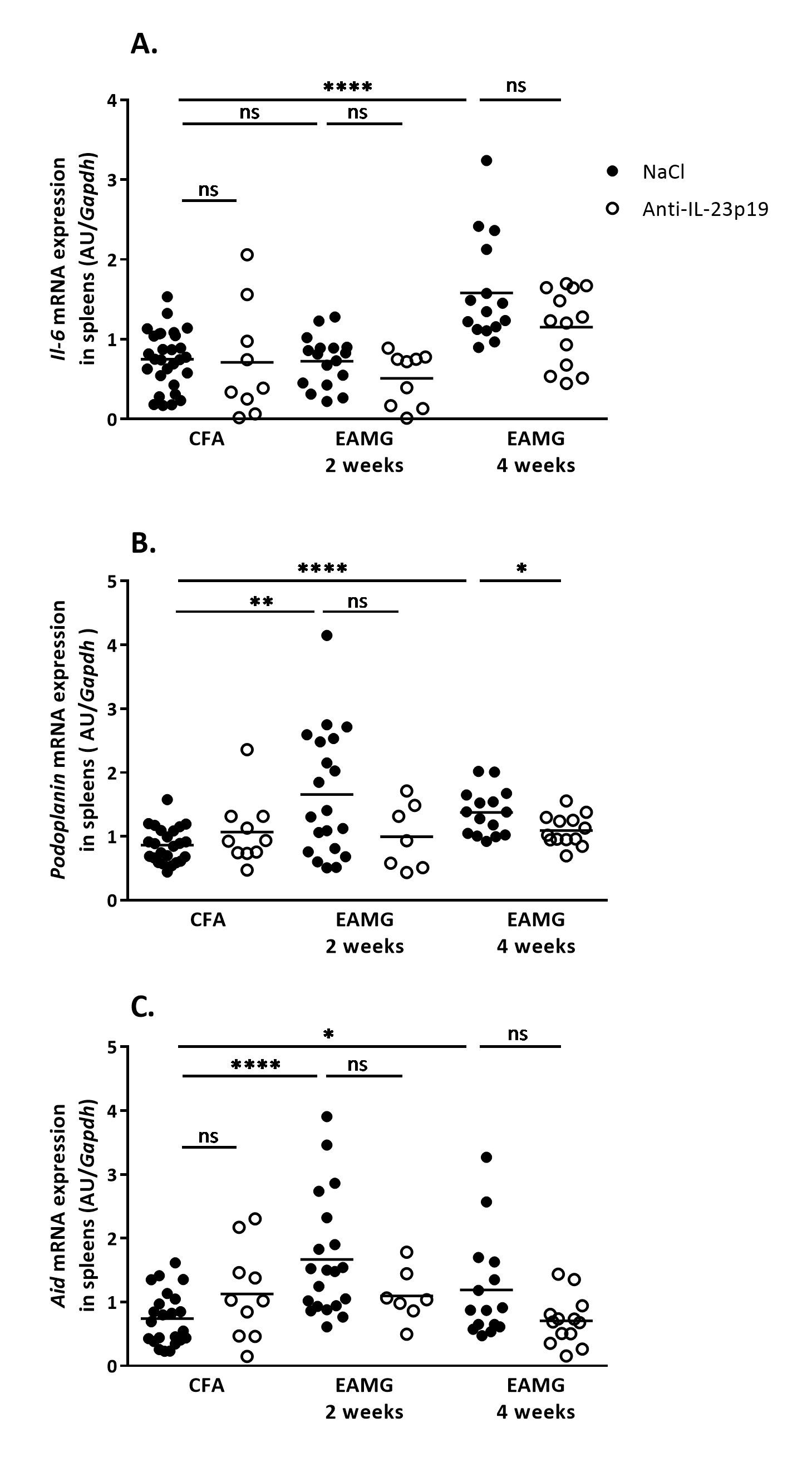
**

**Figure S4. Anti-IL-23p19 tends to reduce markers of eGCs in the spleens of EAMG mice.**

mRNA expression of Il-6 (**A)**, *Podoplanin* **(B)** and *Aid* **(C)** in the spleens of CFA and EAMG mice treated with or without anti-IL-23p19 antibody. mRNA analyses were performed in duplicate after 2 weeks or 4 weeks of treatment by quantitative RT-PCR. For each treatment time, data were obtained from 2 independent experiments. n>4 mice per group. Each point represents an individual mouse. The mRNA results are expressed as arbitrary unit (AU) and normalized to *Gapdh*. P values were obtained with an ANOVA test. P value are as follows *<0.05;**0.004; ****0.0001

**
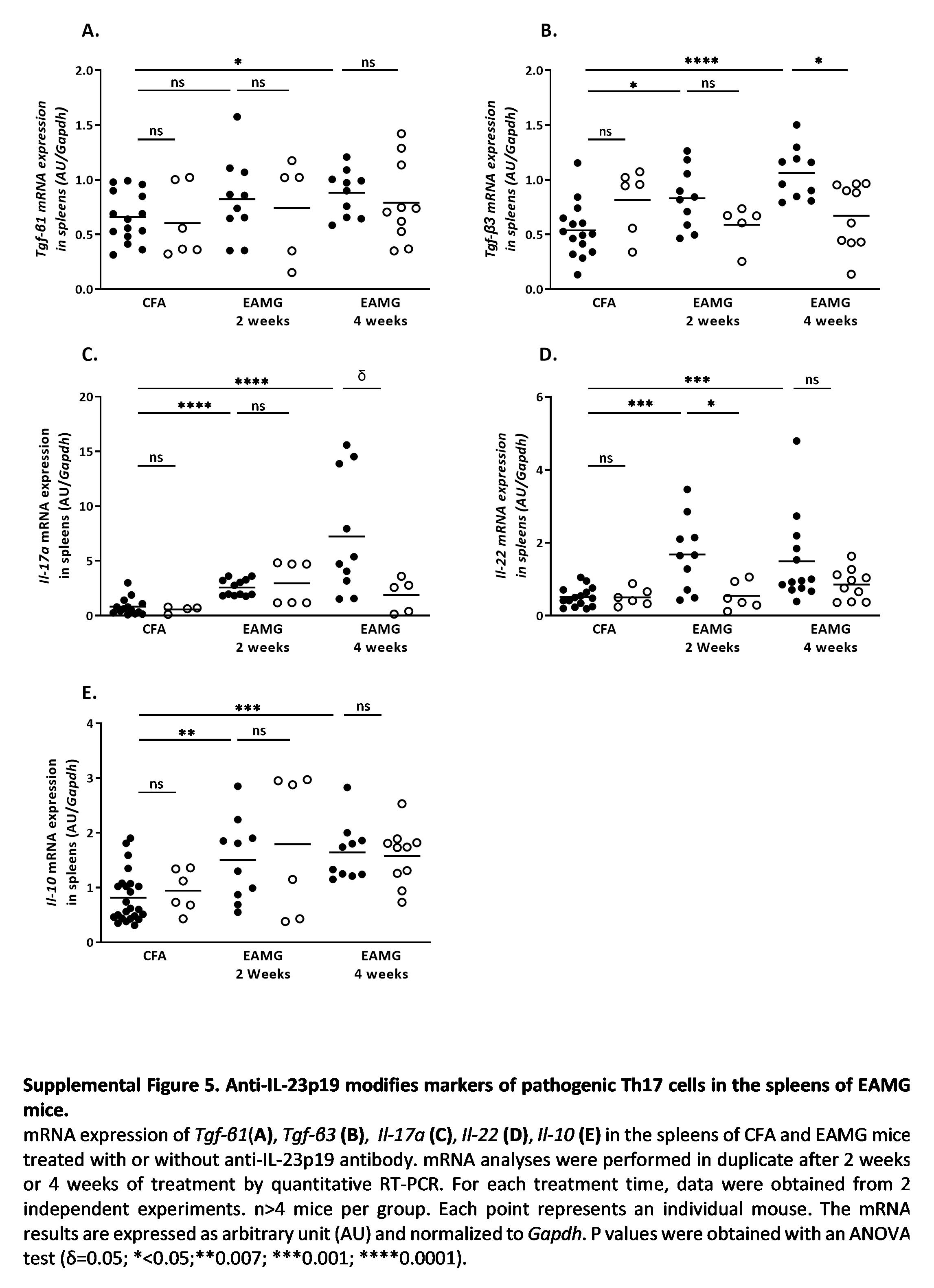
**

**Figure S5. Anti-IL-23p19 modifies markers of pathogenic Th17 cells in the spleens of EAMG mice.**

mRNA expression of *Tgf-β1* (**A)**, *Tgf-β3* **(B)**, *Il-17a* **(C)**, *Il-22* **(D)**, *Il-10* **(E)** in the spleens of CFA and EAMG mice treated with or without anti-IL-23p19 antibody. mRNA analyses were performed in duplicate after 2 weeks or 4 weeks of treatment by quantitative RT-PCR. For each treatment time, data were obtained from 2 independent experiments. n>4 mice per group. Each point represents an individual mouse. The mRNA results are expressed as arbitrary unit (AU) and normalized to *Gapdh*. P values were obtained with an ANOVA test (δ=0.05; *<0.05;**0.007; ***0.001; ****0.0001).
